# Construction Notes

**Published:** 1897-01-16

**Authors:** 


### CONSTRUCTION NOTES.

**Derbyshire Royal Infirmary.—New Wing.**

The new wing at the Royal Infirmary, erected at the expense of Mr. Walter Evans, of Darley Abbey, has been formally opened. The building has cost £12,000, and, being part of the original scheme, was built by Messrs. Walker and Slater from the plans of Messrs. Young and Hall. Consisting of two storeys, there are, downstairs, one ward for twenty-four patients and a small ward for two patients, and, upstairs, a ward for ten patients, a small one for two patients, and four for one each. In addition, there is, upstairs, a small operating theatre. The furnishing of the place and the general appointments are completely in harmony with the rest of the establishment. In all, the new wing provides an additional forty-two beds to an institution which is doing an invaluable work in Derbyshire.

**Extension of Harrogate Cottage Hospital.**

For some time the want of a special ward for children in this hospital has been felt, and a movement was instituted and worked, along with others, to secure the necessary funds for a provision of this kind. It was arranged that it should be named the "Clarence Ward," in honour of the late Duke of Clarence's visit to Harrogate to open the Royal Bath Hospital. Other alterations and additions have since been carried out. The "Clarence Wing" is located at the rear of the building, and gives accommodation for seven children patients. The cost of the new enlargement scheme is about £2,800. The contractors for the ward are Simpson Brothers, Harrogate. Mr. T. E. Marshall, architect of Harrogate, designed the plans.

